# Artificial Flowering Induction Practices in Pineapple (cv. Sugarloaf) Production in Southern Benin: Comparative Analysis of Effectiveness of Induction Agents and Effects on Fruit Quality

**DOI:** 10.1002/pei3.70234

**Published:** 2026-11-13

**Authors:** Orthia L. F. Linkpon, Nicodème V. Fassinou Hotegni, Fernand S. Sohindji, Gildas Houessou, Brunith Ahokpossi, Mouizz A. B. Salaou, Charlotte A. O. Adjé, Enoch G. Achigan‐Dako

**Affiliations:** ^1^ Genetics, Biotechnology and Seed Science Unit (GBioS), Laboratory of Crop Production, Physiology and Plant Breeding (PAGEV), Faculty of Agricultural Sciences University of Abomey‐Calavi Cotonou Republic of Benin

**Keywords:** activated charcoal enriched with ethylene, artificial flowering induction, Benin Republic, calcium carbide, ethephon, pineapple

## Abstract

Flowering Induction Treatment (FIT) is an essential component of the commercial fresh pineapple production system. In Benin Republic and despite the efforts of the government, FIT is still carried out using calcium carbide, which is harmful to human health and the environment. To provide fresh pineapple producers with an eco‐friendly FIT, we assessed the effect of 18 FITs on the flowering emergence and fruit quality parameters of cv. Sugarloaf. FITs consisted of zero application, calcium carbide 1%: 22 kg/ha (widely used), activated charcoal enriched with Ethylene (promoted by the government for organic pineapple production), reduced doses of calcium carbide by 25%–50% plus activated charcoal, fine clay or/and petroleum, and reduced doses of Ethrel plus urea and/or sodium bicarbonate or activated charcoal, and activated charcoal enriched with ethylene. Two experiments were carried out in southern Benin using a randomized complete block design with four repetitions. Results showed that treatments including calcium carbide mixed with activated carbon and clay, and Ethrel mixed with urea and/or bicarbonate, induced flowering in more than 95% of cv. Sugarloaf pineapple plants. With these treatments, the length and weight of pineapple fruit, infructescence, and crown, as well as pH and total soluble solids content, were significantly affected. The number of planting materials was also significantly affected by the FITs. Results indicated that treatments with “Calcium carbide 0.75% + Activated carbon 0.5%” and “Ethrel 200ppm + Urea 4% + Sodium bicarbonate 0.4%” offer interesting prospects for conventional cv. Sugarloaf, in terms of flowering incidence, days to flower, and fruit quality, with potential minimal risks for producers and the environment. The potential implications for producer health, environmental safety, and economic benefits were not directly assessed in this study and should be explored through further investigation.

## 1 Introduction

Climate change is one of the most pressing threats to the world, with many negative effects, raising numerous research questions among researchers and international institutions (FAO 2024; Osman et al. 2024; Williams et al. 2018). Its effects are reported to be worse in African countries with high population vulnerability, rapid demographic growth, and inadequate access to basic needs in water, health, infrastructure, and food and nutrition security (Souad and Yousra 2024). Food and nutrition security is central, as it contributes significantly to a healthier, more stable society (Rosegrant et al. 2024). It implies that all people, at all times, have reliable access to sufficient, safe, and nutritious food necessary for a healthy and active life (Peng and Berry 2019). Nutrition security refers to the consistent access to a variety of nutrient‐dense foods necessary for optimal health and development, taking into account both the quality and quantity of the diet (Peng and Berry 2019). In Sub‐Saharan Africa, food and nutrition security, along with the sanitary security of the harvested product, have been a concern for many years.

During the last decade, stakeholders and policymakers have become more sensitive to the sanitary security of the products they produce and consume (Monacco et al. 2024). This is the case of the Republic of Benin, where the ban of Benin's fresh pineapple fruits in the international market in 2017 (due to non‐compliance with maximum residue limits) brought attention to the need to promote ecological or environmentally friendly practices.

Among the pineapple cultivars produced in Benin, cv. Sugarloaf (locally called Pain de Sucre) is the most grown pineapple cultivar in southern Benin (Fassinou Hotegni et al. 2012). It has a sweet taste, white and juicy flesh, and relatively low acidity (Fassinou Hotegni et al. 2025). Due to the exceptional organoleptic quality of the fruit, cv. Sugarloaf has been registered as a Protected Geographical Indication (PGI) by the African Intellectual Property Organization (OAPI) (Mitchodigni‐Houndoloa et al. 2024), reflecting its recognized geographical identity, quality, and commercial reputation. Given such importance in the Beninese pineapple sector, maintaining or improving fruit quality is particularly relevant for the sustainability and competitiveness of whole cv. Sugarloaf value chains. This will also help to avoid another ban on fresh pineapple from Benin; the previous one was due to non‐compliance with maximum residue levels, a consequence of inadequate agronomic practices used by producers in the field (INSAE 2020). A recent survey indicated that 95% of fresh pineapple producers are still using calcium carbide to induce flowering in pineapple, despite the reported negative effects on their health (Fassinou Hotegni et al. 2025).

In fresh pineapple production, artificial flowering induction of plants, generally called “Flowering Induction Treatment (FIT)” is an essential component of the commercial pineapple production system (Fassinou Hotegni 2014). Such agronomic practice helps reduce temporal variability in flowering and fruit production within a field and allows the synchronization of fruit harvesting (Bartholomew et al. 2003). Over the years, several chemical treatment agents have been developed and/or tested. Among the products used for artificial flowering induction in pineapple plants, the most commonly used are: alpha‐naphthalene acetic acid or its sodium salt (ANA or SNA), beta‐naphthalene acetic acid (BNA), acid 2,4‐dichlorofenoxiacetic (2,4‐D), indole‐butyric acid (IBA), succinic acid, ethylene gas (C_2_H_4_), acetylene (C_2_H_2_), 2‐chloroethane acid phosphonic or Ethephon (Ethrel), calcium carbide (releasing acetylene), hydroxyethylhydrazine (HOH), and b‐hydroxyethylhydrazine (BOH) (Cunha 2005). Unfortunately, most of these products are harmful for both human health and the environment. Calcium carbide (CaC_2_) is widely used for the artificial induction of flowering in pineapple plants in the Republic of Benin (Fassinou Hotegni et al. 2012, 2025) and Togo (Anani et al. 2020). In some countries and regions worldwide, CaC_2_ is even used for fruit ripening (Essien et al. 2018; Lawaly 2022; Oladipupo et al. 2022). Its abusive use and application have been strictly prohibited in many countries due to the multiple health risks associated with both the consumption of artificially ripened fruits and exposure to the product (Lawaly 2022; Oladipupo et al. 2022). In addition to containing traces of phosphorus and arsenic, CaC_2_ in water releases acetylene, a deadly asphyxiant at high acute exposure (Oladipupo et al. 2022). CaC_2_ could weaken the immune system and harm reproductive functions, particularly in men (Essien et al. 2018). Although there is almost no scientific evidence on the sanitary safety of fresh pineapple fruits from plants induced to flower with CaC_2_ (long duration from flowering induction to fruit harvesting: 5 months), its manipulation by fresh pineapple producers could have adverse effects on their health in the short, medium, and long term. Anani et al. (2020) reported that more than 50% of fresh pineapple producers experienced discomfort after applying CaC_2_ solution for the FIT, along with negative environmental impacts, including disruption of biodiversity, soil pollution, and atmospheric pollution. Therefore, there is a need to design sustainable agronomic practices focusing on artificial flowering induction practices for fresh pineapple production with fewer potential effects on producers' health.

In recent years, activated charcoal enriched with ethylene (TIFBio) was developed and promoted within the framework of the COLEACP Pesticides Initiative Program, funded by the European Development Fund (Lebeau et al. 2009). This new product has been promoted in the Republic of Benin since 2020 by the Agricultural Development Territorial Agency (ATDA), which is in charge of expanding fresh pineapple production. Unfortunately, less than 10% of conventional fresh pineapple producers, who produce more than 98% of the pineapple, used it. Such poor use of the TIFBio by fresh pineapple producers is due to its relatively high cost, limited accessibility, and sometimes its effectiveness (Fassinou Hotegni et al. 2025); hence, the urgent need to develop or test new, efficient flowering compound agents for artificial flowering induction. Maruthasalam et al. (2010) showed the possibility of reducing the calcium carbide dose by adding activated charcoal. Moreover, Hepton (2003) discussed the possibility of using fine clay such as bentonite instead of activated charcoal. Dass et al. (1976) suggested reducing the effective concentration of Ethephon (Ethrel) for flowering induction by adding urea to the solution and adjusting the pH to 9.0 with sodium carbonate. However, not only may FITs influence the flowering emergence rate but also the fruit quality. In fresh pineapple production, plant vigor at the time of flowering induction has been associated with variation in fruit weight and other fruit characteristics (Fassinou Hotegni 2014). Furthermore, flowering induction and maturity‐management practices can affect internal (total soluble solids (TSS) and juice pH) and external quality attributes (Fassinou Hotegni et al. 2015b). Therefore, the effects of the FITs on external and internal fruit quality attributes are important.

In sub‐Saharan African countries, very few studies have evaluated the effects of various FITs on pineapple flowering emergence rate and their impact on fruit quality. Therefore, the main objective of this study was to reduce or eliminate the exposure of fresh pineapple producers to the negative effects of CaC_2_ through the proposition of alternatives to CaC_2_ (or a reduction of the quantity used by fresh pineapple producers) without compromising the fruit quality for different markets. Research questions were: what is the effect of different FITs on inflorescence emergence rate and fruit quality? Are Ethrel‐based induction products more effective in inducing inflorescence in pineapple plants than calcium carbide‐based products? To what extent can we reduce the amount of calcium carbide without compromising the effectiveness of the induction and fruit quality? This study is important because it will help fresh pineapple producers produce quality fresh pineapple without being exposed to large amounts of calcium products.

## 2 Materials and Methods

### 2.1 Experimental Sites

Two experiments were conducted in the municipality of Zè, in the Atlantic department in southern Benin, where pineapple is a major crop cultivated by producers (Fassinou Hotegni et al. 2012). Two fields with ferralitic soil located in *Anavié* (Latitude: 6°37′11.6″ N, Longitude: 2°15′31.9″ E) and *Housagoudo* (Latitude: 6°38′25.0″ N, Longitude: 2°16′33.2″ E) were used for Experiment 1 (1600 m^2^) and Experiment 2 (2000 m^2^), respectively. The selection of each experimental field was based on criteria such as the age of the pineapple plants in the field (when the pineapple plants were close to the regular flowering induction phase) and the producer's willingness and consent to host the experiment. Experiment 1 covered the period from March 2023 to December 2023, while Experiment 2 covered the period from July 2023 to January 2024 (Figure 1).

**FIGURE 1:**
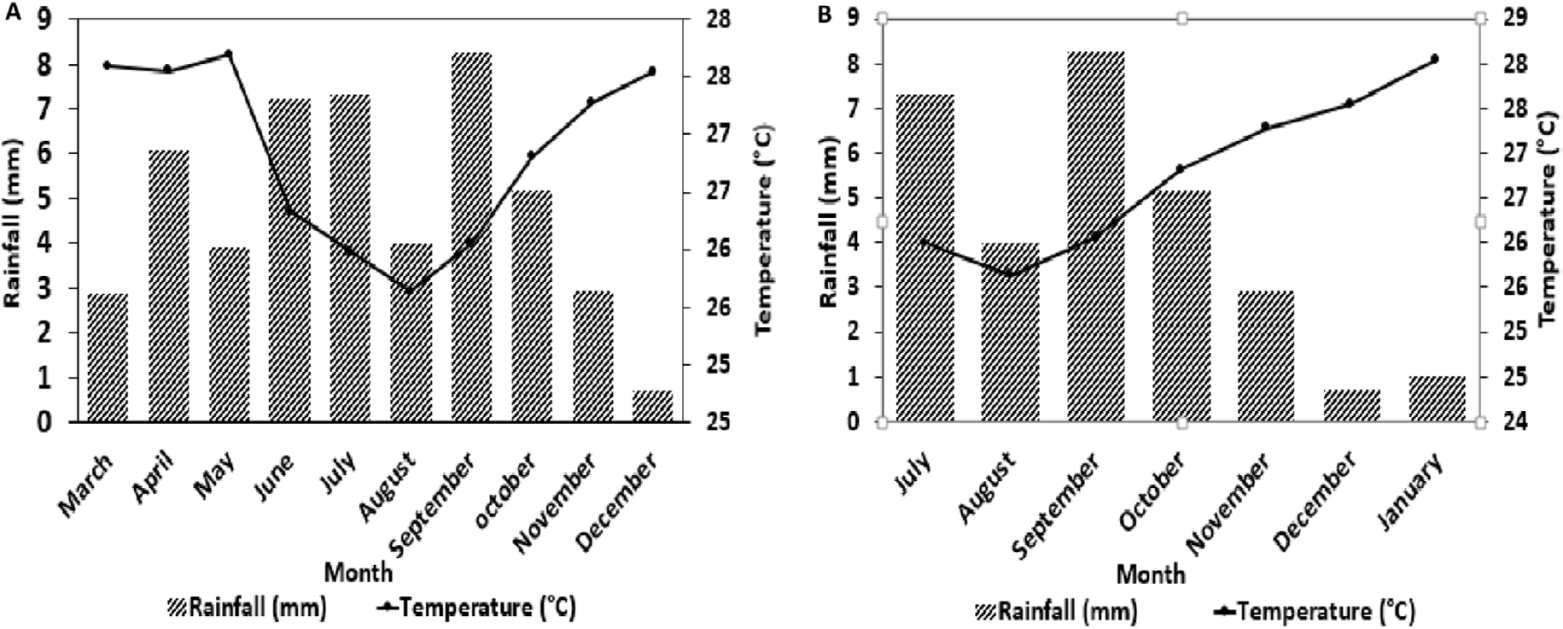
Variation in mean air temperature (°C) and monthly rainfall amount (mm) during Experiment 1 from March to December 2023 (A) and Experiment 2 from July 2023 to January 2024 (B).

Based on the agronomic practices followed at each experimental site, artificial flowering inductions were performed at 13 and 11 months after planting in Experiments 1 and 2, respectively (Table 1).

**TABLE 1:** Description of agronomic practices used on each experimental site.

Agronomic practices	Experiment 1	Experiment 2
Planting time*	April 2022	August 2022
Planting material*	Planting material	Planting material
Planting arrangement	Flat beds of two alternating rows	Flat beds of two alternating rows
Spacing	Between plants within a row: 30 cm Between rows: 40 cm, Between double rows 60 cm, Planting density: 66,666 plants ha^−1^	Between plants within a row: 30 cm Between rows: 40 cm, Between double rows 60 cm, Planting density: 66,666 plants ha^−1^
Fertilizer application before the experiment was set by the researcher*	1 application	2 applications
Application time	6 MAP	3MAP and 6MAP
Type of fertilizer	Urea (46% N)	Urea (46% N) and N_20_P_10_K_10_
Quantity per plant (g)	10	10
Fertilizer application after the experiment was set by the researcher	1 application	1 application
Application time	11 MAP	9 MAP
Type of fertilizer	N_20_P_10_K_10_	N_20_P_10_K_10_ and Urea (46% N)
Quantity per plant (g)	10	10
Manual weed control	Before fertilizer application and artificial flowering induction	Before fertilizer application and artificial flowering induction
Artificial flowering induction time	13 MAP	11 MAP
Harvest time	153 to 160 DAT	156 to 161 DAT

Abbreviations: DAT, Days after artificial flowering induction; MAP, Months after planting.

* Data collected from fresh pineapple producer (field owner).

### 2.2 Experimental Design and Treatments

For each experiment, a randomized complete block design (RCBD) with four blocks and four replications was used. Each experiment initially included 18 treatments (T0–T17), with 24 plants per experimental unit, resulting in a total of 1728 plants per experiment. The control treatments comprised zero application (T0), calcium carbide applied at 1% (22.5 kg ha^−1^; T1), representing the current practices of fresh pineapple producers, and activated charcoal enriched with ethylene (T2), representing the approach promoted by the government for organic fresh pineapple production.

Treatments involving reduced doses of calcium carbide or ethephon, combined with additional compounds, were also evaluated. Reduced calcium carbide doses (25%–50% lower than the standard dose) were combined with either activated charcoal or fine clay (T3–T6). Different ethephon application rates were evaluated (T7), while other treatments combined ethephon with urea (T8–T11) or with urea and sodium bicarbonate (T12–T15). Additionally, ethephon was combined with activated charcoal (T16), and calcium carbide was mixed with oil/petroleum (T17) (Table 2).

**TABLE 2:** Description of artificial flowering induction treatments tested per experiment on cv. Sugarloaf pineapple.

Treatment	Flowering induction compounds	Dose per ha	Per plot (24 plants; 3.6 m^2^)
T0	No application	0 kg	0 g
T1^a^	Calcium carbide 1%	22.5 kg	8.1 g
T2^b^	Activated charcoal enriched with ethylene (TIFBio)	6.99 kg	2.52 g
T3	Calcium carbide 0.5%	11.25 kg	4.05 g
	Activated charcoal 0.5%	11 kg	3.96 g
T4	Calcium carbide 0.75%	16.87 kg	6.08 g
	Activated charcoal 0.5%	11 kg	3.96 g
T5	Calcium carbide 0.5%	11.25 kg	4.05 g
	Fine clay0.5%	11 kg	3.96 g
T6	Calcium carbide 0.75%	16.875 kg	6.08 g
	Fine clay0.5%	11 kg	3.96 g
T7	Ethrel 500 ppm	1.5 L	0.54 mL
T8	Ethrel 300 ppm	0.99 kg	0.36 mL
	Urea 4.5%	113.51 kg	40.86 g
T9	Ethrel 200 ppm	0.66 L	0.24 mL
	Urea 4%	100.90 kg	36.32 g
T10	Ethrel 100 ppm	0.333 L	0.12 mL
	Urea 3%	63.06 kg	22.7 g
T11	Ethrel 50 ppm	0.165 L	0.06 mL
	Urea 2%	50.45 kg	18.16 g
T12	Ethrel 300 ppm	0.999 L	0.36 mL
	Urea 4.5%	113.517 L	40.86 mL
	Sodium bicarbonate0.4%	0.6 kg	0.22 g
T13	Ethrel 200 ppm	0.666 L	0.24 mL
	Urea 4%	100.9 kg	36.32 g
	Sodium bicarbonate 0.4%	0.6 kg	0.22 g
T14	Ethrel 100 ppm	0.333 L	0.12 mL
	Urea 3%	63.06 kg	22.7 g
	Sodium bicarbonate 0.4%	0.6 kg	0.22 g
T15	Ethrel 50 ppm	0.165 L	0.06 mL
	Urea 2%	50.452 kg	18.16 g
	Sodium bicarbonate 0.4%	0.6 kg	0.22 g
T16	Ethrel 200 ppm	0.666 kg	0.24 mL
	Activated charcoal 0.5%	11 kg	3.96 g
T17^c^	Calcium carbide 0.75%	16.875 kg	6.08 g
	Petroleum	2 L	0.7 mL

^a^ Fresh pineapple producers' practice.

^b^ Artificial Induction practice being introduced by the government.

^c^ Practice already adopted by certain producers.

### 2.3 Preparation and Application of Flowering Induction Treatments

The flowering induction treatments were prepared separately and used in every four experimental units receiving the same treatment across the four blocks. Each plant received 50 mL of the prepared solution. The quantities of the treatment compound are specified in Table 2. Basically, treatment compounds were introduced into a clean container, followed by the addition of clean, cooled water. The mixture was homogenized before application. After completing the application of a given treatment across the four blocks, the container was thoroughly washed before preparing the next treatment to minimize cross‐contamination between treatments. For treatments combining calcium carbide (CaC_2_) with activated charcoal or fine clay, the activated charcoal or clay was first placed in the container, followed by the addition of the required quantity of clean, cooled water. The mixture was stirred thoroughly before calcium carbide was added. The container was then closed, and the preparation was left for the reaction to take place before application. For the treatment combining calcium carbide with petroleum, the calcium carbide and petroleum were placed together in a container, which was then closed and shaken repeatedly to mix the components. No adjuvant was added to this preparation.

For the Ethrel‐based treatments, the order of preparation depended on the treatment composition. In the Ethrel–urea–sodium bicarbonate treatment, urea was first dissolved in water, after which sodium bicarbonate was added and mixed before Ethrel was introduced last. For the Ethrel–urea treatment, urea was dissolved in water before Ethrel was added. For the treatment combining Ethrel with activated charcoal, the charcoal was introduced before Ethrel. The flowering induction treatments were applied once at the center of the leaf rosette, using 50 mL of the prepared solution. A homemade backpack, commonly used by fresh pineapple producers in Benin, was used to apply the different FITs. Applications were made in the evening at the center of the leaf rosette because stomata on pineapple plants open at night and could facilitate the absorption of gases from the compound applied (Bartholomew et al. 2003). No rain occurred within 24 h following the treatment.

### 2.4 Data Collection

Collected data included plant vigor, inflorescence emergence, external and internal fruit quality, and production of planting material.

Data on plant vigor were collected 3 weeks before flowering induction and included D‐leaf length and the number of functional leaves, as described by Fassinou Hotegni (2014).

$$\text{Plant vigour}=\text{Dleaf length}\;\left(\mathrm{dl}\right)\times \text{number of functional leaves}\;\left(\mathrm{nl}\right)$$

The *D‐leaf length (dl)* was measured on the longest leaf among all the leaves of the plant. A 40 cm twig was used to mark the first 40 cm from the base of the leaf. A yellow wooden ruler was used to measure the remaining length beyond these 40 cm. The *number of functional leaves on the plant (nl)*, representing the number of functional leaves, was determined manually.

Data on inflorescence emergence rate were collected after flowering induction. They included the number of days from flowering induction to inflorescence emergence and the number of plants that flowered each day. Inflorescence emergence was characterized by the appearance of the red heart, characterized by the visible reddish coloration of the center of the leaves rosette, as described by Fassinou Hotegni et al. (2015a). The number of days from artificial flowering induction to inflorescence emergence was recorded by counting the days from the time the artificial FITs were applied until inflorescence emergence at each plant. The number of plants that had flowered was recorded at 5‐day intervals, from 30 to 75 days after each FIT (30, 35, 40, 45, 50, 55, 60, 65, 70, and 75 days), to systematically monitor flowering progression.

External (length of fruit, infructescence, and crown; weight of fruit, infructescence, and crown) and internal (pH, Total Soluble Solids content) fruit quality, as well as planting material (called slips and which develop just below the fruit on cv. Sugarloaf) production data were collected at harvesting time. Data on the infructescence length, crown length, and fruit length were collected using a flat graduated ruler. The weight of the fruit, infructescence, and crown was recorded using an electronic balance.

To determine fruit juice pH and Total Soluble Solids (TSS) content, each fruit was cut longitudinally and divided into three sections: the base, middle, and apex. Juice was extracted from each section, pooled in a container, and thoroughly mixed to obtain a homogeneous sample representative of the entire fruit. The TSS content of the pooled juice was measured using a refractometer, while juice pH was determined using a pH meter (Dass et al. 1976; Kumari et al. 2020; Maruthasalam et al. 2010; Raposo et al. 2019; Sabando‐Ávila et al. 2023; Selamat et al. 2005).

### 2.5 Data Analysis

Data analysis was carried out using R software version 4.3.0 (R Core Team 2023). It included analysis of variance (ANOVA), linear mixed‐effects model with a covariate, a generalized linear Poisson model with mixed effects, and Tukey post hoc and LSD tests. ANOVA was used to assess the effect of treatments on data related to plant vigor and flowering emergence data at 75 DAT. A linear mixed‐effects model with a covariate was applied to the juice pH and TSS data, with Treatment as a fixed factor, Block as a random factor, and plant vigor as a covariate. In addition, the marginal and conditional R^2^ of the linear mixed‐effects models were calculated using the *r*‐squared GLMM () function of the MuMIn package (Barton 2016). A generalized linear Poisson model with mixed effects was applied to the counting data of the number of planting materials. Tukey's and/or LSD tests were used for the means separation at the 5% significance level.

Although 18 treatments were initially planned, the number of treatments retained for analysis was subsequently reduced to 17 for Experiment 2 because T17 was incorrectly applied to the plants by the workers. Consequently, T17 was excluded from the combined statistical analysis of Experiments 1 and 2, while it was retained in the analysis of Experiment 1, where applicable. The treatment levels included in each analysis are specified in the corresponding tables.

## 3 Results

### 3.1 Effect of Artificial Flowering Compounds on Inflorescence Emergence in cv. Sugarloaf Pineapple Plants

Results indicated a very low inflorescence emergence rate in the control T0 treatment (< 10%). High inflorescence emergence (> 90%) was observed in the other controls, T1, and T2 at 75 DAT in both experiments. In Experiment 1, some experimental units reached 50% inflorescence emergence around 45 DAT (38–45 DAT), whereas in Experiment 2, the 50% flowering in some experimental units was earlier, around 35 DAT (17–37 DAT). Regarding the alternative treatments, the total inflorescence emergence rate at the end of the experiments ranged from 85% to 100% after 75 DAT. However, the highest values of the inflorescence emergence rate were observed with the treatments based on reduced doses of calcium carbide T3 (95%), combined with activated charcoal T4 (100%), or fine clay T5 (100%) and T6 (100%), or petroleum T17 (95%) (Figure 2). Highest values of the inflorescence emergence rate were also reported for treatments based on reduced doses of Ethrel combined with urea or urea + bicarbonate, including T8 (100%), T9 (100%), T10 (98%), T11 (98%), T12 (98%), T13 (100%), T14 (98%), T15 (100%), and T16 (98%) compared to Ethrel 500 ppm treatment T7 (98%) (Figure 3).

**FIGURE 2:**
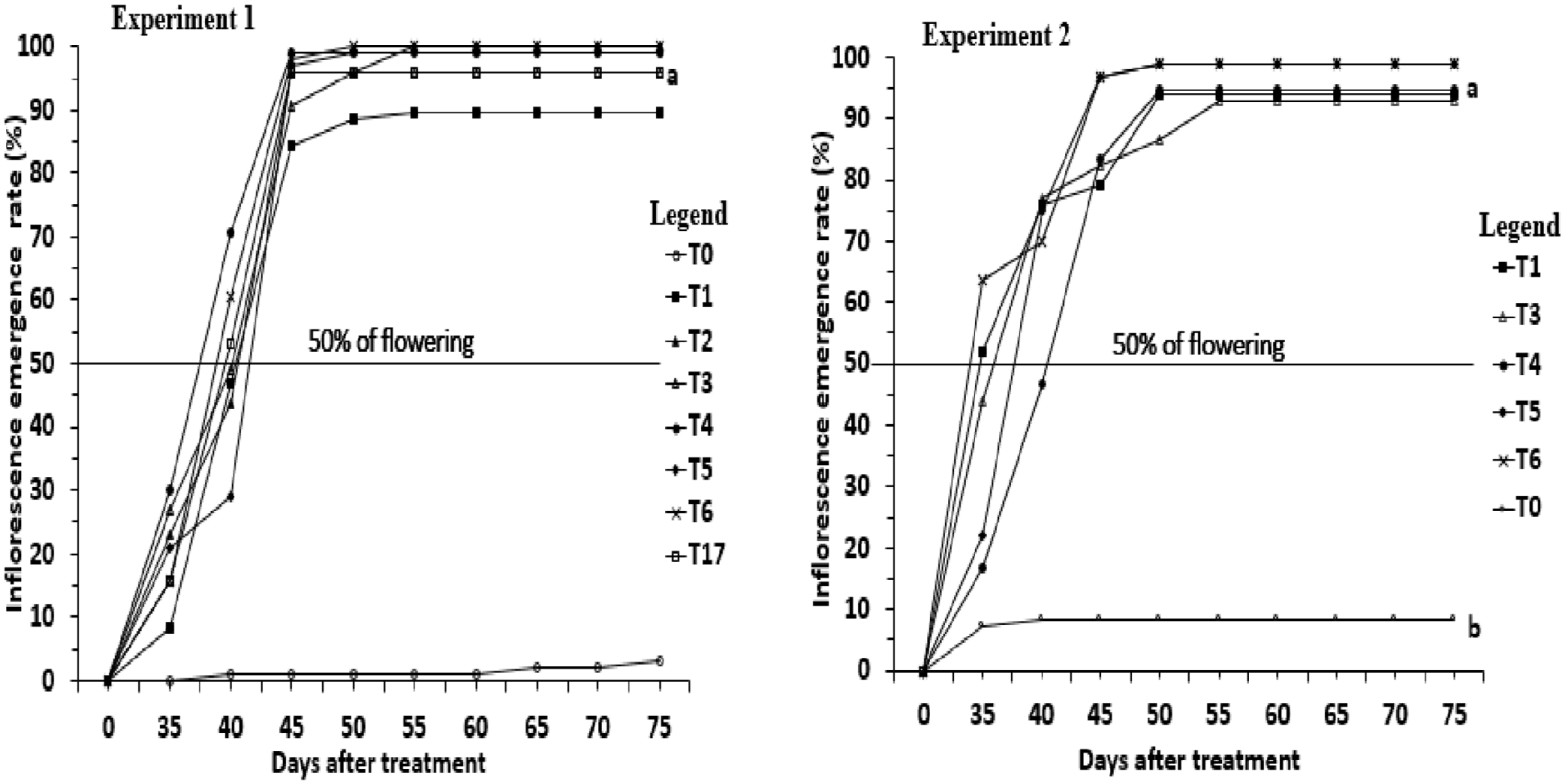
Evolution of the inflorescence emergence rate on sugarloaf plants treated with calcium carbide‐based compounds (T3, T4, T6, T17) from 35 to 75 days after flowering induction compared to the control treatments (T0, T1, T2). Each symbol on the graph (for each line) represents the means of the four replicates. T0: No application, T1: Calcium carbide (CaC_2_ 1%); T2: Activated charcoal enriched with ethylene; T3: CaC_2_ 0.5% + Activated charcoal 0.5%; T4: CaC_2_ 0.75% + Activated charcoal 0.5%; T5: CaC_2_ 0.5% + Fine clay 0.5%; T6: CaC_2_ 0.75% + Fine clay 0.5%; T17: Calcium carbide 0.75% + Petroleum.

**FIGURE 3:**
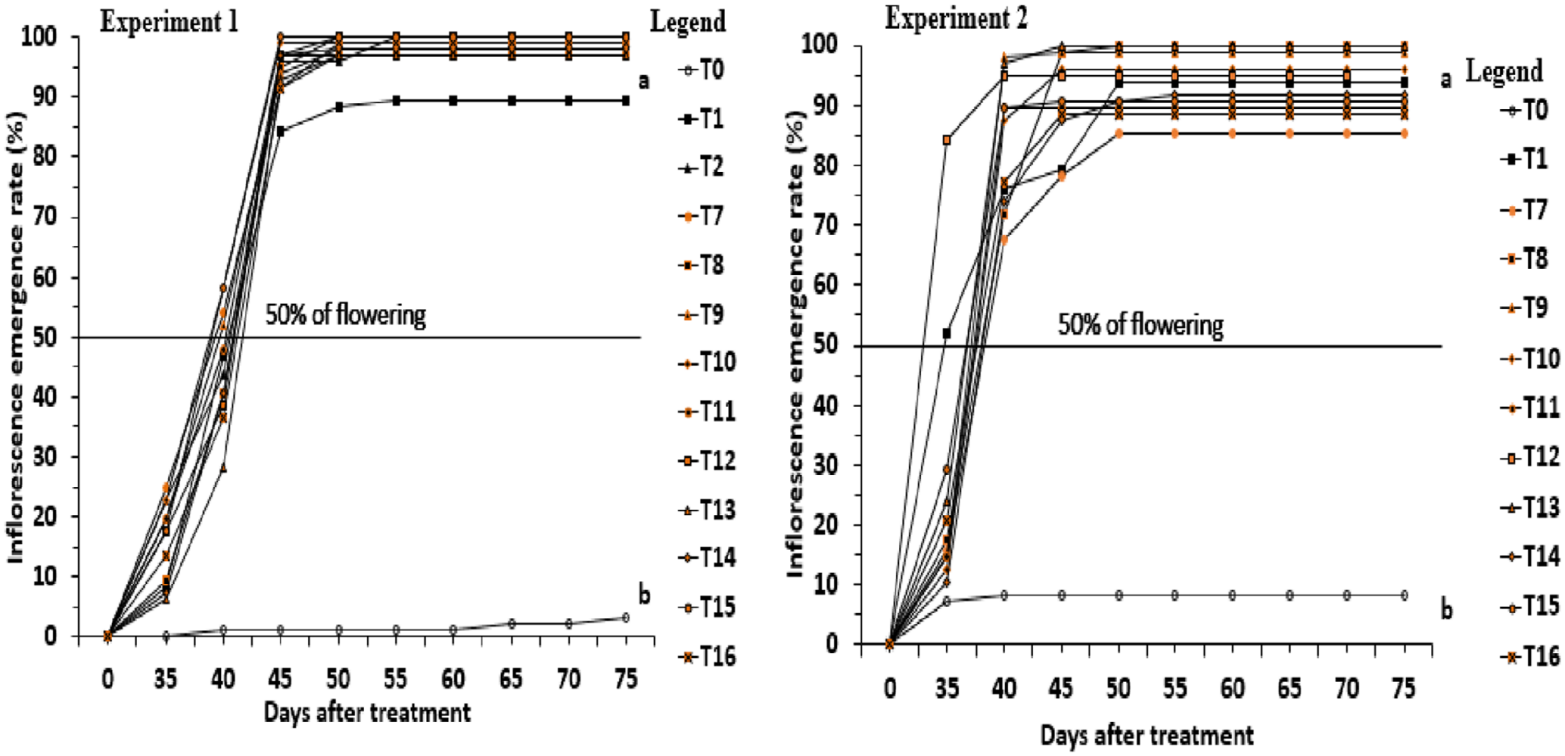
Evolution of the inflorescence emergence rate on pineapple plants treated with Ethrel‐based compounds (T7, T8, T9, T10, T11, T12, T13, T14, T15, T16) from 35 to 75 days after flowering induction compared to the control treatments (T0, T1, T2). Each symbol on the graph (for each line) represents the means of the four replicates. T0: No application, T1: Calcium carbide 1%; T2: Activated charcoal enriched with ethylene; T7: Ethrel 500 ppm, T8: Ethrel 300 ppm + Urea 4.5%; T9: Ethrel 200 ppm + Urea 4%; T10: Ethrel 100 ppm + Urea 3%; T11: Ethrel 50 ppm + Urea 2%; T12: Ethrel 300 ppm + Urea 4.5% + Sodium bicarbonate 0.4%; T13: Ethrel 200 ppm + Urea 4% + Sodium bicarbonate 0.4%; T14: Ethrel 100 ppm + Urea 3% + Sodium bicarbonate 0.4%; T15: Ethrel 50 ppm + Urea 2% + Sodium bicarbonate 0.4%; T16: Ethrel 200 ppm + Activated charcoal 0.5%.

In both experiments, the analysis of variance showed significant differences in flowering emergence rate between the different artificial flowering induction techniques and the control T0 (*p*‐value < 0.001). At the same time, there was no significant difference (*p*‐value = 0.53) between the alternative treatments and the producer practices T1 (Table 3).

**TABLE 3:** *p*‐values and *R*‐squared from the analysis of variance (ANOVA) comparing artificial flowering induction treatments against the control (T0) and the producer's practice (T1).

Inflorescence emergence rate (%) at 75 days after artificial flowering induction	Experiment 1 (T0–T17)		Experiment 2 (T0–16)	
	*p*	*R* ^2^	*p*	*R* ^2^
Treatments against T0	0.001**	0.9588	0.001**	0.9214
Treatments against T1	0.53	0.227	0.07	0.3533

*Note: R* ^2^: multiple *R*‐squared, T0: No application, T1: Calcium carbide.

** Statistically significant at *p* < 0.001.

### 3.2 Effects of Different Artificial Flowering Induction Treatments on Fresh Pineapple Fruit Quality Attributes

The effect of different artificial flowering induction treatments (T0–T16) and plant vigor (NL × DL) on fruit attributes, including length (total fruit, infructescence, and crown) and weight (fruit, infructescence, and crown) assessed (Table 4).

**TABLE 4:** Effect of artificial flowering induction treatments (T0–T16) and plant vigor (NL × DL) on sugarloaf pineapple total fruit, infructescence, and crown attributes, including length and weight.

Variables	Sources of variation	NumDF	DF	*F*‐statistic	*p*
Total fruit length (cm)	Treatment	16	215	2.69	0.0006671***
	Plant vigor (NL × DL)	1	191	53.22	7.738e^−12^ ***
Infructescence length (cm)	Treatment	16	222	2.63	0.0008623***
	Plant vigor (NL × DL)	1	222	92.47	< 2.2e^−16^ ***
Crown length (cm)	Treatment	16	203	3.58	1.048e^−05^ ***
	Plant vigor (NL × DL)	1	153	2.36	0.1264
Total fruit weight (g)	Treatment	16	218.01	3.88	2.107e^−06^ ***
	Plant vigor (NL × DL)	1	205.00	135.84	< 2.2e^−16^ ***
Infructescence weight (g)	Treatment	16	217.04	3.84	2.645e^−06^ ***
	Plant vigor (NL × DL)	1	200.35	142.47	< 2.2e^−16^ ***
Crown weight (g)	Treatment	16	54.62	7.38	1.02e^−08^ ***
	Plant vigor (NL × DL)	1	32.604	4.55	0.0405*

Abbreviations: DF, Degrees of freedom in the denominator related to the overall variation of the model; NL × DL, number of functional leaves × the D‐leaf length standing for plant vigor; NumDF: Degrees of freedom in the numerator related to fixed factors.

* *p*‐value < 0.05.

*** *p*‐value < 0.001.

Results showed that the artificial FITs, as well as the plant vigor (NL × DL) at artificial flowering induction time, contributed significantly to the total fruit length, infructescence length, and crown length (only the artificial flowering induction treatments). Across the two experiments, plants induced with compound T13 (Ethrel 200 ppm + urea 4% + sodium bicarbonate 0.4%) produced fruits with the highest average total length and infructescence length (40.12 and 22.27 cm, respectively) and a shorter crown (17.85 cm) (Figure 4).

**FIGURE 4:**
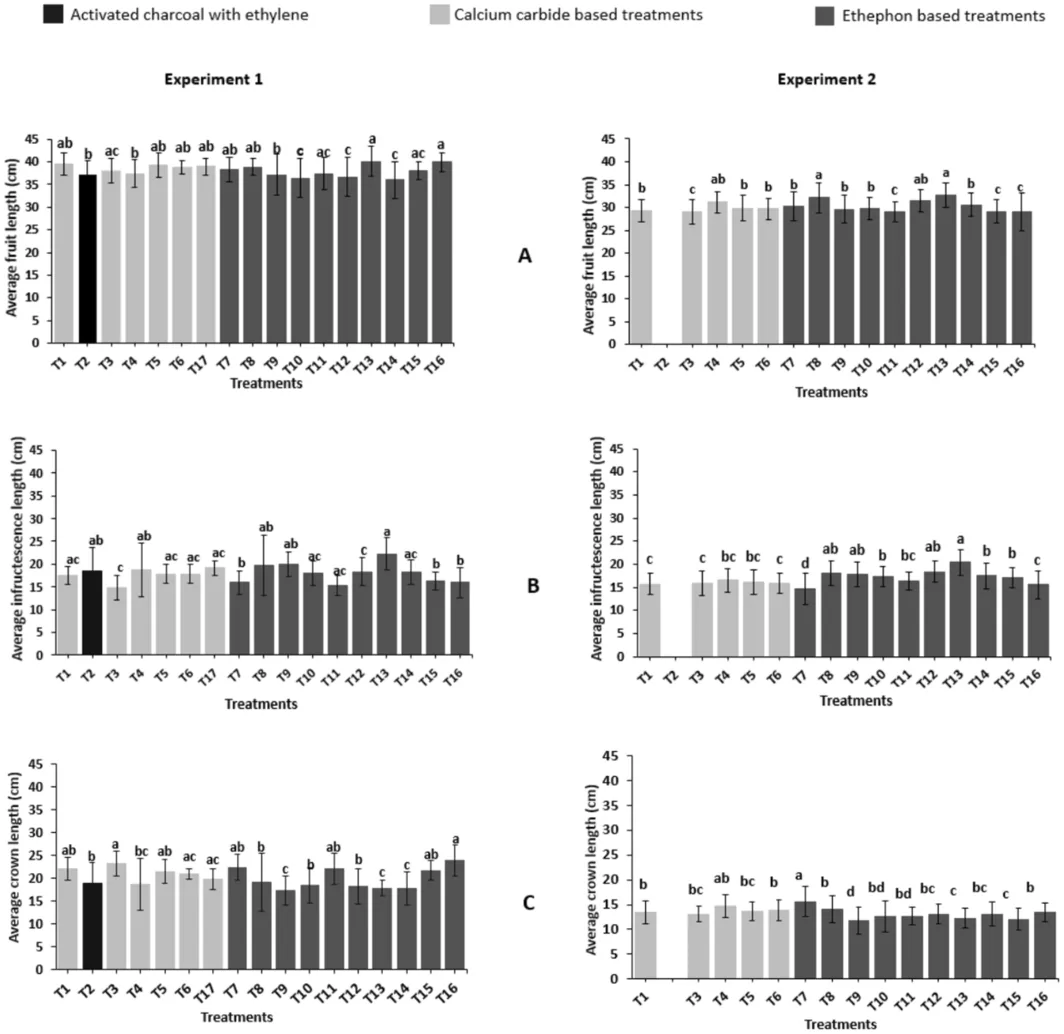
Effects of different artificial flowering induction treatments on sugarloaf pineapple total fruit length (A), infructescence length (B), and crown length (C). Plots with the same letter are not significantly different, while plots with different letters are significantly different. T0: No application, T1: Calcium carbide 1%; T2: Activated charcoal enriched with ethylene; T3: CaC_2_ 0.5% + Activated charcoal 0.5%; T4: CaC_2_ 0.75% + Activated charcoal 0.5%; T5: CaC_2_ 0.5% + Fine clay 0.5%; T6: CaC_2_ 0.75% + Fine clay 0.5%; T7: Ethrel 500 ppm, T8: Ethrel 300 ppm + Urea 4.5%; T9: Ethrel 200 ppm + Urea 4%; T10: Ethrel 100 ppm + Urea 3%; T11: Ethrel 50 ppm + Urea 2%; T12: Ethrel 300 ppm + Urea 4.5% + Sodium bicarbonate 0.4%; T13: Ethrel 200 ppm + Urea 4% + Sodium bicarbonate 0.4%; T14: Ethrel 100 ppm + Urea 3% + Sodium bicarbonate 0.4%; T15: Ethrel 50 ppm + Urea 2% + Sodium bicarbonate 0.4%; T16: Ethrel 200 ppm + Activated charcoal 0.5%; T17: Calcium carbide 0.75% + Petroleum.

The total fruit weight, infructescence weight, and crown weight were also affected significantly by the artificial flowering induction treatments and plant vigor at the time of artificial flowering induction. Across the two experiments, plants induced with compound T13 (Ethrel 200 ppm + urea 4% + sodium bicarbonate 0.4%) produced fruits with the highest average total weight and infructescence weight (1537.9 and 1425.4 g, respectively) and the lightest crown (Figure 5).

**FIGURE 5:**
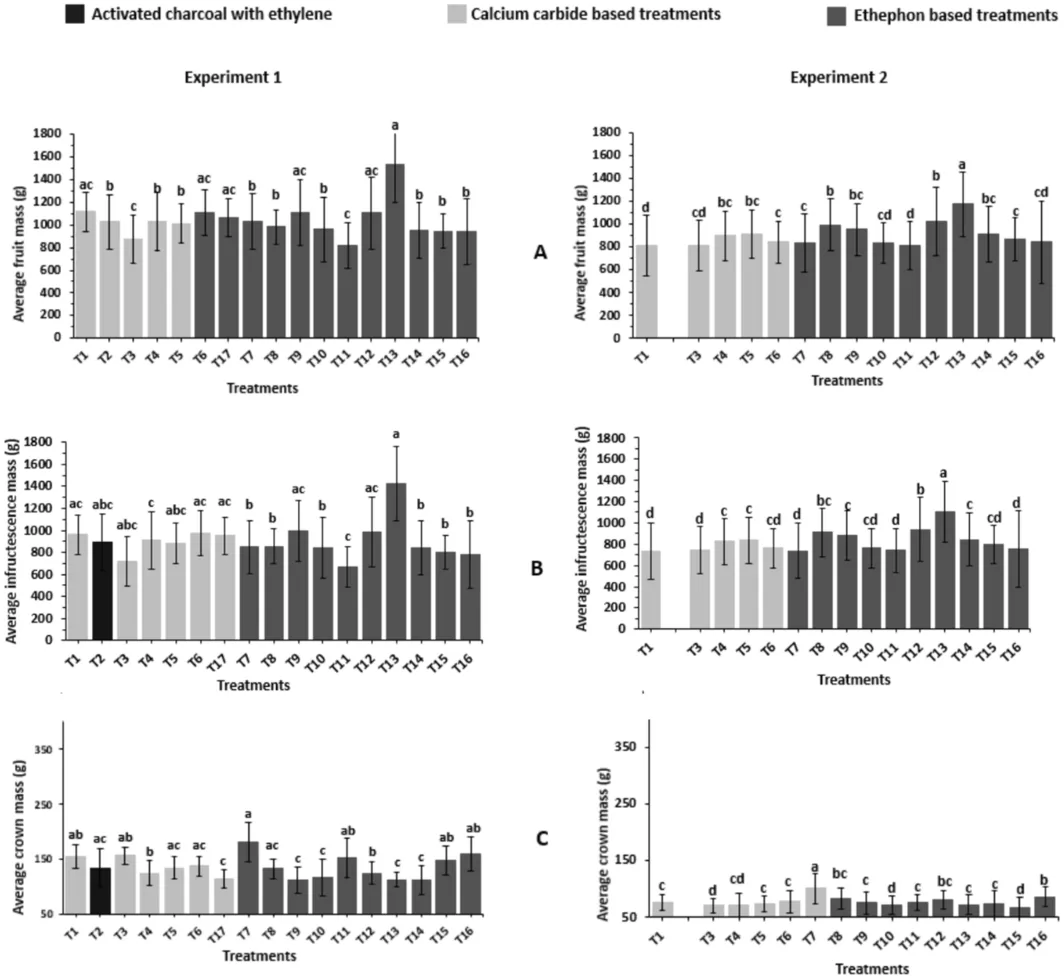
Effects of different artificial flowering induction treatments on sugarloaf pineapple total fruit weight (A), infructescence weight (B) and crown weight (C). Plots with same letter are not significantly different while plots with different letters are significantly different. T0: No application, T1: Calcium carbide 1%; T2: Activated charcoal enriched with ethylene; T3: CaC_2_ 0.5% + Activated charcoal 0.5%; T4: CaC_2_ 0.75% + Activated charcoal 0.5%; T5: CaC_2_ 0.5% + Fine clay 0.5%; T6: CaC_2_ 0.75% + Fine clay 0.5%; T7: Ethrel 500 ppm; T8: Ethrel 300 ppm + Urea 4.5%; T9: Ethrel 200 ppm + Urea 4%; T10: Ethrel 100 ppm + Urea 3%; T11: Ethrel 50 ppm + Urea 2%; T12: Ethrel 300 ppm + Urea 4.5% + Sodium bicarbonate 0.4%; T13: Ethrel 200 ppm + Urea 4% + Sodium bicarbonate 0.4%; T14: Ethrel 100 ppm + Urea 3% + Sodium bicarbonate 0.4%; T15: Ethrel 50 ppm + Urea 2% + Sodium bicarbonate 0.4%; T16: Ethrel 200 ppm + Activated charcoal 0.5%.

### 3.3 Effect of Different Artificial Flowering Induction Treatments on Internal Fruit Quality

The different artificial flowering‐induction compounds applied to induce flowering in pineapple plants, and the plant vigor at the time of flowering induction, had a highly significant effect on the pH of fresh pineapple juice and TSS (Table 5).

**TABLE 5:** Effect of different artificial flowering treatments (T0–T16) and plant vigor (NL × DL) at the artificial flowering induction time on the pH and TSS of fresh sugarloaf pineapple juice.

Variable	Sources of variation	NumDF	DenDF	*F*‐statistic	*p*
pH	Treatment	16	222	5.9596	8.072e^−11^ ***
	Plant vigor (NL × DL)	1	222	13.9619	0.0002373***
Total Soluble Solids (°Brix)	Treatment	16	217	1.8866	0.02289*
	Plant vigor (NL × DL)	1	217	4.9104	0.02774*

Abbreviations: DenDF, Degrees of freedom in the denominator related to the overall variation of the model; NL × DL, number of functional leaves × the D‐leaf length standing for plant vigor; NumDF, Degrees of freedom in the numerator related to fixed factors.

* *p*‐value < 0.05.

*** *p*‐value < 0.001.

The highest average pineapple juice pH value (3.67) was obtained with the treatment T16: Ethrel 200 ppm + Activated charcoal 0.5%, and the lowest value (3.42) with the treatment T17: Calcium carbide 0.75% + Petroleum 17 in the first experiment. In Experiment 2, the highest average pineapple juice pH value (4.06) was obtained with the treatment T3: Calcium carbide 0.5% + Activated charcoal 0.5%, and T4: Calcium carbide 0.75% + Activated charcoal 0.5%, and the lowest value (3.96) with the treatment T7: Ethrel 500 ppm.

The average TSS values obtained vary between 13.09°Brix (T10: Ethrel 100 ppm + Urea 3%) and 14.34°Brix (T5: Calcium carbide 0.5% + Fine clay 0.5%) in Experiment 1 and 13.52°Brix (T15: Ethrel 50 ppm + Urea 2% + Sodium bicarbonate 0.4%) and 14.75 Brix (T4: Calcium carbide 0.75% + Activated charcoal 0.5%) in Experiment 2. The means separation analysis revealed two clusters (a and b) for pH and three clusters (a, b, and c) for TSS, which are significantly different (Figure 6).

**FIGURE 6:**
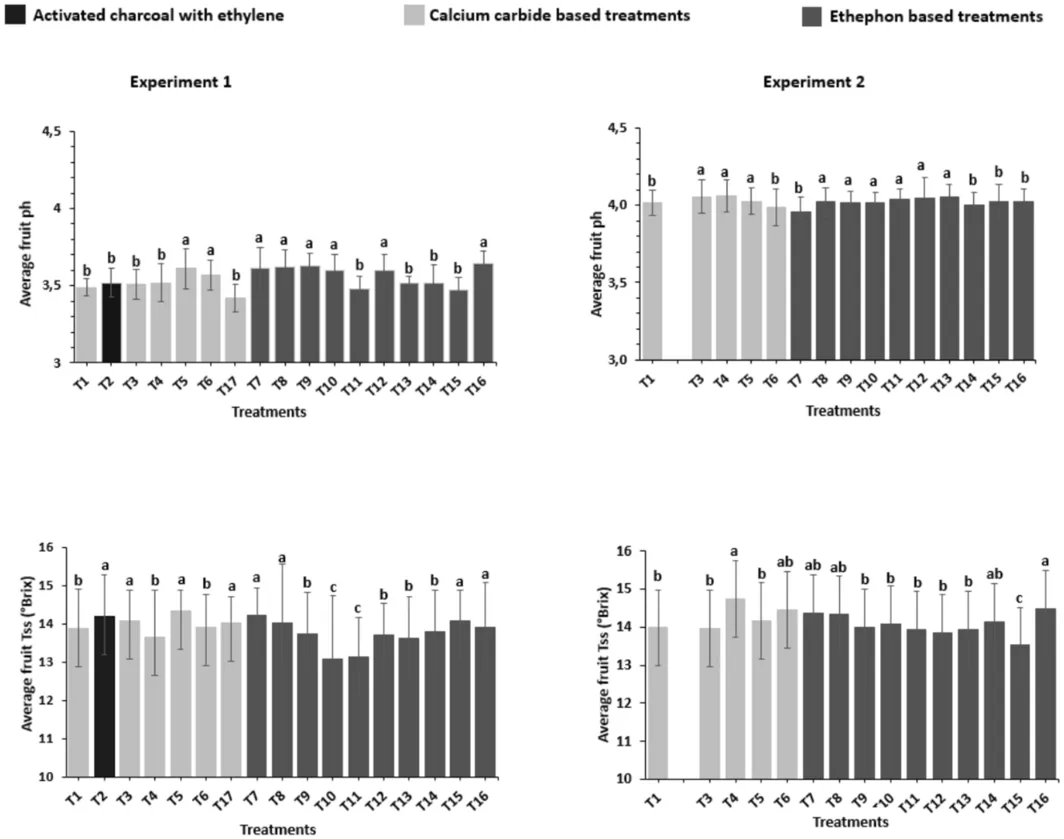
Variation in pH and Total Soluble Solid content of sugarloaf pineapple juice induced by flowering induction treatments. Plots with the same letter are not significantly different while plots with different letters are significantly different. T0: No application, T1: Calcium carbide 1%; T2: Activated charcoal enriched with ethylene; T3: CaC_2_ 0.5% + Activated charcoal 0.5%; T4: CaC_2_ 0.75% + Activated charcoal 0.5%; T5: CaC_2_ 0.5% + Fine clay 0.5%; T6: CaC_2_ 0.75% + Fine clay 0.5%; T7: Ethrel 500 ppm, T8: Ethrel 300 ppm + Urea 4.5%; T9: Ethrel 200 ppm + Urea 4%; T10: Ethrel 100 ppm + Urea 3%; T11: Ethrel 50 ppm + Urea 2%; T12: Ethrel 300 ppm + Urea 4.5% + Sodium bicarbonate 0.4%; T13: Ethrel 200 ppm + Urea 4% + Sodium bicarbonate 0.4%; T14: Ethrel 100 ppm + Urea 3% + Sodium bicarbonate 0.4%; T15: Ethrel 50 ppm + Urea 2% + Sodium bicarbonate 0.4%; T16: Ethrel 200 ppm + Activated charcoal 0.5%.

### 3.4 Effects of Different Artificial Flowering Induction Treatments on the Planting Material Produced by the Pineapple Plants

Results indicated that artificial flowering induction treatments had a significant effect on the number of planting material produced by the plant (*p*‐value < 0.001), with the lowest number reported with treatment T7 (Ethrel 500 ppm) (Figure 7).

**FIGURE 7:**
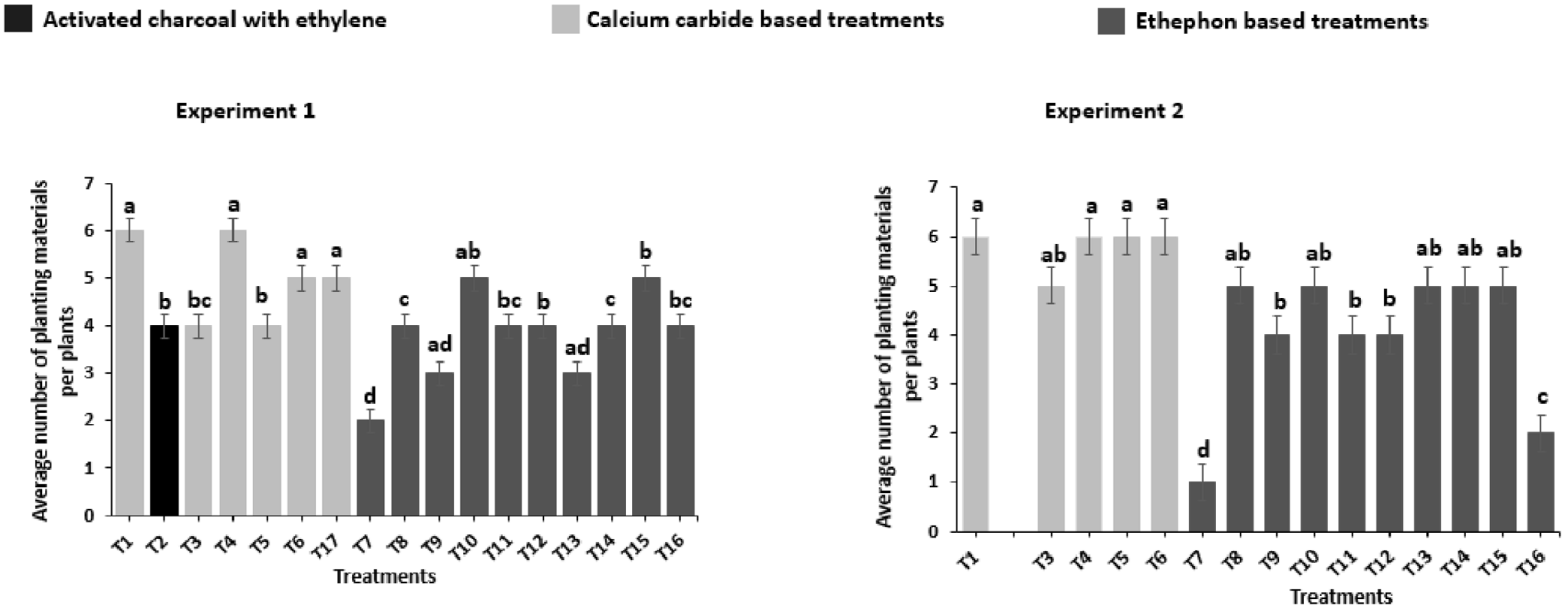
Variation in planting material production of sugarloaf pineapple induced by flowering induction treatments. Plots with the same letter are not significantly different while plots with different letters are significantly different. T0: No application, T1: Calcium carbide 1%; T2: Activated charcoal enriched with ethylene; T3: CaC_2_ 0.5% + Activated charcoal 0.5%; T4: CaC_2_ 0.75% + Activated charcoal 0.5%; T5: CaC_2_ 0.5% + Fine clay 0.5%; T6: CaC_2_ 0.75% + Fine clay 0.5%; T7: Ethrel 500 ppm, T8: Ethrel 300 ppm + Urea 4.5%; T9: Ethrel 200 ppm + Urea 4%; T10: Ethrel 100 ppm + Urea 3%; T11: Ethrel 50 ppm + Urea 2%; T12: Ethrel 300 ppm + Urea 4.5% + Sodium bicarbonate 0.4%; T13: Ethrel 200 ppm + Urea 4% + Sodium bicarbonate 0.4%; T14: Ethrel 100 ppm + Urea 3% + Sodium bicarbonate 0.4%; T15: Ethrel 50 ppm + Urea 2% + Sodium bicarbonate 0.4%; T16: Ethrel 200 ppm + Activated charcoal 0.5%; T17: Calcium carbide 0.75% + Petroleum.

In both experiments, the treatments that resulted in the highest number of planting material were T4 (CaC_2_ 0.75% + Activated charcoal 0.5%), T6 (CaC_2_ 0.75% + Fine Clay 0.5%), T1 (Calcium Carbide (CaC_2_)), T17 (CaC_2_ 0.75% + Petroleum), and T5 (CaC_2_ 0.5% + Fine Clay 0.5%), while the lowest number of planting material was obtained with Ethrel‐based treatments including T7 (Ethrel 500 ppm), T9 (Ethrel 200 ppm + Urea 4%), T13 (Ethrel 200 ppm + Urea 4% + Sodium bicarbonate 0.4%), T14 (Ethrel 100 ppm + Urea 3% + Sodium bicarbonate 0.4%), and T12 (Ethrel 300 ppm + Urea 4.5% + Sodium bicarbonate 0.4%).

## 4 Discussion

This study aimed to identify alternative pineapple flowering treatments aligned with the transition to eco‐friendly practices in pineapple production in African countries, focusing on flowering and fruit quality parameters (external and internal) of cv. Sugarloaf pineapple under 18 flowering induction techniques were assessed in southern Benin. Treatments were formulated as combinations of compounds, including calcium carbide‐based solution, activated charcoal enriched with Ethylene, and Ethephon (Ethrel). No study has documented the effect of the addition of these mixtures on the effectiveness of flowering induction or pineapple fruit quality. This study provided effective alternative techniques promoting inflorescence emergence and fresh fruit quality in the pineapple cv. Sugarloaf.

### 4.1 Effect of Artificial Flowering Compounds on Inflorescence Emergence in cv. Sugarloaf Pineapple Plants

Our results clearly indicated that the different compounds, including calcium carbide‐based solution, activated charcoal enriched with Ethylene, and ethephon, increased by more than 80% the flowering ability of pineapple cv. Sugarloaf compared to the control T0 (no application). However, some compounds have proven to be more effective than others.

#### 4.1.1 Effect of Calcium Carbide and Activated Charcoal Enriched With Ethylene

The T1 treatment, based only on calcium carbide corresponding to producers' practice, produced on average an inflorescence emergence of 84.38% at 45 days after treatment, reaching almost 90% at 55 days after treatment. The treatment T2, standing for activated charcoal enriched with ethylene in suspension, induced almost 96% flowering at 45 DAT and 100% at 55 DAT. This indicates the effectiveness of activated charcoal enriched with ethylene in suspension compared to calcium carbide treatment, as reported by Van De Poel et al. (2009) in Cameroon (89% flowering) and in Martinique (100% flowering) with activated charcoal enriched with ethylene in suspension. Lebeau et al. (2009) also obtained up to 100% flowering at 56 DAT with ‘TIFBio’ in cv. Smooth Cayenne cultivation in Togo, the best case for use in suspension. These two compounds induced hormonal changes in pineapple by releasing ethylene or similar compounds, such as acetylene (Bartholomew et al. 2003). Ethylene gas is a natural plant growth regulator that activates signaling pathways that induce a series of physiological responses, including flowering (Eckert et al. 2014). It is produced in a two‐step reaction from methionine via S‐adenosyl‐methionine (SAM). SAM is first converted to 1‐aminocyclopropane‐1‐carboxylic acid (ACC) by ACC synthase, and ACC oxidase catalyzes the oxidative release of ethylene and cyanide (CN) (Mathur et al. 2023). As a substitute for ethylene, acetylene (from calcium carbide) acts through an indirect pathway. When used in flowering treatments, acetylene breaks down into ethylene, which contributes to its effectiveness, but this conversion is not as immediate or direct as using ethylene itself. Additionally, the preparation of the carbide solution generates a significant amount of heat, which could potentially lead to additional ethylene production alongside the acetylene produced by the reaction of calcium carbide with water. Activated charcoal enriched with ethylene exploits the adsorption properties of activated carbon as a vector for gaseous ethylene (Lebeau et al. 2009). Here, the activated carbon is stored in polypropylene containers, allowing ethylene to be conserved for long periods without mass loss due to outgassing. All these facts may explain why activated charcoal is much more effective than calcium carbide.

#### 4.1.2 Effect of Reduced Doses of Calcium Carbide Mixed With Other Products

All carbide treatments at reduced doses showed effectiveness in inducing flowering in pineapple plants. The most important effects were obtained with treatments based on a 25% reduction in calcium carbide, including T4 (Calcium carbide 0.75% + Activated charcoal 0.5%), T6 (Calcium carbide 0.75% + Fine Clay 0.5%), and T17 (Calcium carbide 0.75% + Petroleum). Inflorescence emergence ranged from 60.4% to 70.83% at 40 DAT and reached 98% to 100% at 45 DAT. These results support those of Maruthasalam et al. (2010), who demonstrated that CaC_2_ solutions (0.5 or 1.0%) supplemented with activated charcoal (0.5%) significantly improved the CaC_2_ forcing efficiency in flowering induction. This result indicated the possibility of reducing the usual carbide amount by up to 50% by adding simple activated charcoal or fine clay to promote floral induction in pineapple. This can be explained by the high absorbency of activated charcoal, which helped limit acetylene loss for as long as possible, a phenomenon that generally occurs during the disintegration of CaC_2_ in water (Maruthasalam et al. 2010). Fine clay also has better absorption characteristics, leading to this prolonged retention of acetylene, and resulting in a prolonged availability of this gas at the level of the apical meristem of pineapple plants. Petroleum could potentially form a surface film that alters gas exchange within the leaf rosette and affects the retention or escape of gases released by the flowering induction treatment. However, this explanation remains hypothetical because gas exchange and ethylene concentrations within the rosette were not measured in the present study. Further investigations are therefore needed to determine whether petroleum influences flowering induction through such physical effects or other mechanisms.

#### 4.1.3 Effect of Reduced Doses of Ethrel Mixed With Urea or/and/or Bicarbonate

The ethrel treatments performed as flowering induction agents. The first flowering signals, consisting of a red turn in the heart of the plant and the appearance of a bud, were mainly observed first in the Ethrel treatments. After 50 DAT, 91% to 97% flowering was reported for the application of Ethrel alone at 500 ppm (T7), 97% to 100% for Ethrel + urea (T8–T11), 98% to 100% for Ethrel + urea + sodium bicarbonate (T12–T15), and 91% to 98% for Ethrel 200 ppm + Activated charcoal 0.5% (T16) (Figure 4). This result indicated that adding urea or sodium bicarbonate at reduced doses to Ethrel improved flowering performance compared with Ethrel alone. The effective action of ethephon is reported to be influenced by adding urea or increasing the pH of the ethrel solution (Bartholomew et al. 2003; Cunha 2005). According to Dass et al. (1976), urea facilitated greater ethephon absorption by the plant. In contrast, the addition of (Na)_2_CO_3_ or CaCO_3_ increased the pH of the solution to 9.0, enabling maximum release of ethylene to the plants. The addition of urea also aligns with a strategy used by producers to reduce the paid workforce by combining urea application with flowering‐induction treatment.

### 4.2 Effects of Different Artificial Flowering Induction Treatments on Fresh Pineapple Fruit Quality Attributes and Planting Material Production

Results showed that activated charcoal enriched with ethylene, calcium carbide, and ethephon‐based treatments induced changes in fresh fruit quality parameters, including total fruit weight and length, infructescence weight and length, crown weight and length, pH, TSS, and the number of planting material. The number of planting material and the length and weight of the crowns were significantly reduced with activated charcoal compared to calcium carbide treatment. The fruits collected from plants treated with Ethrel alone were shorter and lighter, with larger crowns, fewer planting materials, and more defects, compared to the simple carbide treatment. Contrary to these results, Selamat et al. (2005) reported zero effect of floral induction techniques using calcium carbide, Ethephon, and/or ANA (Alpha‐naphthalene acetic acid) on the fresh fruit quality parameters of Josepine pineapple. Ethylene is known to influence plant height after its absorption, acting as a gibberellin antagonist (Hayashi et al. 2001). He showed that the stem height of daffodils and hyacinths can be reduced by using Ethephon. Ethephon would have an antagonistic effect on plant growth and, by extension, on fruit development, leading to the production of small fruits. We can argue that the more ethylene the flower induction treatment involves, the more growth parameters, such as fruit length and weight, will be negatively affected.

However, alternative treatments based on reduced doses of calcium carbide or ethephon, with additional compounds, showed significant results on fresh fruit quality. Treatment T3 (CaC2 0.5% + activated charcoal 0.5%) led to slightly smaller fruits than those obtained with the control T1 (CaC_2_ 1%), whereas T4 (CaC_2_ 0.75% + activated charcoal 0.5%) maintained the same number of planting material while reducing the crown length and weight compared to the control T1 (CaC_2_ 1%). Results highlighted the treatment with Ethrel 200 ppm + urea 4% + sodium bicarbonate 0.4% (T13) as yielding high‐length, heavy fruits, with the highest average infructescence length (40.12 cm and 22.27 cm, respectively) and a shorter crown (17.85 cm), but with reduced number of planting material. Generally, we noted that the addition of urea resulted in larger fruits, larger infructescence, and smaller crowns in the treatment where it was added. Previous studies reported the importance of nutrient fertilizers on the pineapple fresh fruit quality. Urea, as a nitrogen‐rich compound, provides essential nutrients for plant growth (Espinosa et al. 2017). Omotoso and Akinrinde (2013) reported the positive effect of urea on fresh pineapple fruit size as well as its negative effect on the TSS and pH of fresh pineapple fruits. Pineapple fruit yield components, including fruit length, weight, and diameter, must increase with increasing rates (> 150 kg) of N application while decreasing fruit TSS, pH, and Vitamin C (Omotoso and Akinrinde 2013). It is therefore plausible that the amount of urea added in this treatment was sufficient to compensate for the inhibitory effects of Ethephon on plant development, thereby improving fruit development. In addition, severe leaf burn at the heart of the plant was observed in pineapple plants that received treatments containing urea, particularly with high doses of urea. This aligns with the results of Wang et al. (2023), who reported leaf burns at high urea doses. In this study, its severity and incidence were not systematically quantified. Further studies should therefore evaluate phytotoxicity using standardized scoring methods to determine the extent of leaf damage and its implications for plant health and the practical suitability of these treatments.

Furthermore, from a commercial quality perspective, mean total soluble solids (TSS) values in both experiments (13.09°–14.34°Brix in Experiment 1 and 13.52°–14.75°Brix in Experiment 2) exceeded the minimum threshold of 12°Brix specified in the Codex Standard for Pineapples (CXS 182–1993), indicating that all FITs gave pineapple fruits meeting this maturity criterion.

Juice pH varied significantly among treatments, ranging from 3.42 to 3.67 in Experiment 1 and from 3.96 to 4.06 in Experiment 2. These values fall within the range reported for pineapple fruit (Fassinou Hotegni et al. 2015b), although pH varies with cultivar, maturity, and growing conditions (de Ancos et al. 2017). However, the commercial significance of the observed pH differences cannot be established from pH measurements alone, as consumer perception of sweetness and acidity depends on the balance between soluble solids and titratable acidity. Sensory evaluation would therefore be required to determine whether these differences translate into meaningful changes in eating quality and consumer acceptance.

### 4.3 Contribution of Environment and Plant Vigor to the Studied Trait Variation

Results indicated that other factors, including environmental conditions and plant vigor at the time of flowering induction, may affect the efficiency of FITs. Floral induction with Ethrel is a fairly widespread practice in tropical countries, but less common in intertropical countries where climatic variation is low and plant growth is rapid (Teisson 1979). Some growers abandoned its use due to obtaining shriveled fruit (Fassinou Hotegni et al. 2025). The earlier flowering response reported in Experiment 2 may be due to water stress (lack or infrequency of rain) during the flowering induction period, compared to Experiment 1, where rainfall increased. It is known that water stress increases ethylene biosynthesis, thereby enhancing plant flowering (Bartholomew et al. 2003). Plant vigor at the time of flowering induction influenced the morphological and weight parameters of fruit and infructescence. Previous studies reported that variation in plant vigor can result in heterogeneity in fruit weight (Fassinou Hotegni 2014). However, pH and total soluble solids content of fruit seem to be affected by plant vigor. Here, two alternative flower induction treatments, including T4 (CaC_2_ 0.75% + activated charcoal 0.5%) and T13 (Ethrel 200 ppm + urea 4% + sodium bicarbonate 0.4%), showed satisfactory effectiveness in terms of flowering emergence rate, days to flower, and fruit quality produced with low potential risks for the environment and farmers.

## 5 Conclusion

This study demonstrates that alternative flowering induction treatments can effectively induce flowering in Sugarloaf pineapple while influencing fruit physical and chemical quality. The findings indicate that both calcium carbide‐based and Ethrel‐based formulations offer potential alternatives to conventional induction practices, although their relative effectiveness depends on the formulation and application rate. Reducing calcium carbide application by 25% in combination with activated charcoal showed promise for flowering emergence and fruit quality while reducing the amount of calcium carbide applied. However, the observed leaf burn associated with high urea concentrations warrants caution when considering Ethrel–urea formulations for practical use. Although minor leaf burn was observed following the application of the Ethrel–urea treatment, the affected plants recovered within approximately 2 weeks. Moreover, this treatment produced larger fruits in both experiments, suggesting that the observed leaf damage did not visibly prevent subsequent fruit development under the conditions of this study. Nevertheless, as phytotoxicity was not systematically quantified, further assessment is needed to confirm the extent and implications of this response before making broader recommendations for farmer adoption.

## Funding

This work has been funded by the Genetics, Biotechnology, and Seed Science Unit (GBioS) at the Faculty of Agricultural Sciences, University of Abomey‐Calavi, Republic of Benin.

## Conflicts of Interest

The authors declare no conflicts of interest.

## Data Availability

The datasets generated and analyzed during the current study are available in the Figshare repository: https://doi.org/10.6084/m9.figshare.33339081.
